# Bone mineral density changes during treatment of rheumatoid arthritis with disease-modifying-anti-rheumatic drugs 

**Published:** 2012

**Authors:** Behzad Heidari, Mahmoud Monadi, Mohammad Ali Ghazi Mirsaed

**Affiliations:** 1 Department of Internal Medicine, Ayatollah Rouhani Hospital, Babol University of Medical Sciences, Babol, Iran.

**Keywords:** Rheumatoid arthritis, BMD, DMARD therapy, Corticosteroids.

## Abstract

**Background:**

Bone mineral density (BMD) changes during the course of rheumatoid arthritis (RA). The present study was designed to investigate the status of BMD in patients with RA treated with anti-rheumatic drugs.

**Methods:**

BMD at the femoral neck (FN-BMD) and lumbar spine (LS-BMD) were measured by dual energy x-ray absorptiometry (DXA) method using Norland densitometer. Disease activity (DA) was assessed by calculation of DAS28 score. The patients with at least twice BMD measurements were included and those who received treatment for osteoporosis were excluded. The mean FN-BMD and LS-BMD changes from baseline between the two BMD measurements was determined.

**Results:**

Nineteen patients (17 females, 2 males) with the mean age of 54.5±7.7 years, with mean disease duration of 141.8±58 months were treated for an average period of 2.9±1.9 years. All the patients were treated with low-dose methotrexate (MTX) up to 15 mg/week alone or with combination of hydroxychloroquine and/or sulfasalazine and 5 mg prednisolone daily. At the end of study period, the value of FN-BMD gr/cm2 decreased by - 4.24% (p=0.12) and LS-BMD gr/cm2 by - 6.57% (p=0.009). The mean FN BMD Z-score increased by +7.66% (p=0.64) and LS-BMD Z-score decreased by - 14.7% (p=0.120).

**Conclusion:**

The findings of this study indicate that bone loss in RA continues despite anti-inflammatory treatment. The lower rate of bone loss from FN compared with LS may be attributed to suppression of hip synovitis with anti-inflammatory treatment.

Inflammatory process in rheumatoid arthritis (RA) causes bone loss, osteoporosis (OP) and increased risk of bone fractures (1, 2). RA is associated with both localized and generalized osteoporosis. Localized osteoporosis can be considered to be caused by local disease mechanisms such as activation of the cytokines pathway (1). Pro-inflammatory cytokines secreted by the immune cells affect osteoblastic as well as osteoclastic cells and consequently result in increased bone resorption and decreased bone formation (2). In addition, inflammation leads to changes in biomechanical properties of bone and alterations in bone components through increased production of pro-inflammatory cytokines or by hormone mediated mechanisms. Increased production of pro-inflammatory cytokines such as interlukine-1, tumor necrosis factor- alfa. and interlukine-6 are contributing factors of bone loss particularly at the time of menopause (2, 3). These cytokines activate osteoclastic bone resorption. Several factors such as disease duration, disease activity (DA), and functional impairment may independently contribute of each other to bone loss especially in the proximal femur. However, functional impairment is the result of joint destruction or erosions due to inflammation (4, 5). Therefore radiographic damages in RA are related with generalized bone loss (6, 7). 

However, despite the multifactorial nature of bone loss in RA, inflammation has a major contributing role in the development as well as the progression of bone loss in these patients as more severe diseases are associated with greater risk of bone loss. Hence, the greater level of bone loss at the femoral neck in RA should be attributed to increased level of inflammation and turnover at the hip joint (8). Anti-inflammatory treatment decreases bone resorption. This issue was shown in a study of RA patients who were treated with DMARDs. 

In this study, two years of treatment with DMARDs decreased the levels of bone resorption markers such as deoxypyridinoidine (D-PYR) significantly (9). Consequently, suppression of inflammatory process in RA is expected to preserve further bone mass by decreasing DA. On the other hand, anti-inflammatory treatment with corticosteroids may be associated with bone loss due to cumulative dose of steroids (10, 11). 

These observations indicate that anti-inflammatory treatment including corticosteroids through counteracting the inflammatory process and preventing joint destruction can improve functional impairment and preserve further bone mass (2, 12). For these reasons the present study was designed to determine the status of BMD in RA patients treated with anti-rheumatic drugs. 

## Methods

Diagnosis of RA was confirmed by the 1987 American College of Rheumatology revised criteria (13). The BMD at the femoral neck (FN-BMD) and lumbar spine (L2-L4) regions (LS-BMD) were measured by dual energy x-ray absorptiometry (DXA) method using Norland densitometere. DA was assessed by calculation of DAS28 utilizing ESR, swollen and tender joint counts (the 28-joint count), and the patients assessment of pain on a visual analogue scale (14). All the patients were treated with low-dose MTX up to 15 mg/week alone or with the combination of hydroxychloroquine and/or sulfasalazine. Almost all the patients received 5 mg prednisolone daily. Drugs doses were adjusted to reduce DAS28 to lower than 3.2 or achieve clinical improvement or disease remission. Patients who had two time BMD measurements with at least one - year interval were included in the study and those patients who received treatment for osteoporosis were excluded The objective of this study was to determine the mean FN-BMD and LS-BMD changes from baseline between the two BMD measurements. In statistical analysis ,the mean differences in BMD gr/cm2 and BMD Z-score between the two measurement periods were determined at the FN and LS. Independent t-test was used to compare the quantitative valuables and chi-square test was used to compare the qualitative variables. SPSS version 18 was used for data analysis. 

## Results

Nineteen patients (17 females, 2 males) with the mean age of 54.5±7.7 years, with mean disease duration of 141.8±58 months, were studied. Seventeen out of 19 patients were anti-CCP positive and 15 out of 19 were RF positive, and 15 out of 19 were positive for both antibodies (table 1). The initial BMD measurement was performed on 8.3±6.4 years after the onset of RA (median, 6.5 years, range 1- 24 years) and the second BMD measurements was performed after a mean duration of 2.9±1.9 years (median duration 2.5 years, range 1-7 years) of treatment. 

**Table 1:** Characteristics of patients (N=19)

Females / males (NO)	17/2
age (Mean ±SD) (years)	54.5±7.7
Disease duration (months) (mean±SD)	141.8±58
Duration of treatment ( years) (mean±SD)	2.9±1.9
Anti-CCP positivity No (%)	17 (89.4)
Rheumatoid factor positivity.No (%)	15 (19)

The femoral neck BMD gr/cm^2^ decreased from 0.73 (0.11) gr/cm^2^ at baseline (before the first BMD measurement) to 0.70 (0.12) gr/cm^2^ (-4.24%). The mean ± SE difference from baseline was -0.031±0.01, (95%CI -0.008- 0.07, p= 0.11). In anti-CCP positive RA the mean difference from baseline was (-5.34%) -0.039±0.017, p=0.041. In rheumatoid factor positive RA the mean femoral neck BMD decreased from baseline was 0.048±0.018 (6.57%), p=0.020. 

The spine BMD decreased from 0.90±0.16 at baseline to 0.84±0.17 gr/cm^2^ (-6.57%). The mean±SE difference was 0.058±0.19 (95% CI, 0.016-0.10, p=0.009). In Anti-CCP positive RA the mean±SE difference was 0.05±0.021 (5.55%) (p=0.031) and in rheumatoid factor positive patients the mean±SE difference was -0.057±0.023, (6.33%) p=0.028. The mean femoral neck BMD Z-score increased from -1.07±92 at baseline ( before treatment) to -0.99±0.95 (+7.66%), mean±SE difference +0.082±0.17, (95% CI, -0.28-0.45, p=0.643). The mean spine BMD Z-score decreased from -1.02± 0.92 to -1.18±89 (-14.7%), mean±SE difference -0.15±0.09 (0.95% CI, -0.36-0.045, p=0.120) (table 2).

**Table 2:** Changes in femoral neck (FN)and lumbar spine (LS) bone mineral density compared with baseline values in rheumatoid arthritis patients treated with anti-rheumatic drugs

**Site of BMD**	**Baseline**	**End point**	**Mean differences** ** (% Change) (95%CI)**
Femoral neck	0.73±0.11	0.70±0.12	-0.031±0.01 (-4.24 %) (95% CI, -0.008-0.07, p=0.11)
lumbar spine (-6.57%)	0.90±0.16	0.84±0.17	-0.058±0.19 (-6.57 %) (95% CI, 0.016-0.10, p=0.009)
FN-BMD Z-score	-1.07±0.92	-0.99±0.95	0.082±0.17 (+7.66 %) (95% CI, -0.28-0.45, p=0.643)
LS-BMD Z-score	-1.02±0.92	-1.18±89	-0.15±0.09 ( 1.4.7%) (95% CI, -0.36-0.045, p=0.120)

## Discussion

The findings of this study indicated that during treatment of RA with DMARD in combination with low-dose prednisolone, the femoral neck BMD decreased non-significantly by 4.24% and spine BMD decreased significantly by 6.5% compared with baseline values. The mean differences were greater in seropositive patients. However, the comparison of BMD Z-score bone mass before and after treatment demonstrated a nonsignificant increase in femoral neck BMD Z –score by 7.6%, whereas, at the lumbar spine BMD Z –score decreased non significantly by 14.7%. These findings indicate that bone loss in RA patients continues despite DMARD therapy. However, in this study similar to most previous studies, the magnitude of changes from baseline values attributed to RA itself could not be determined due to lack of control group. The results of this study are comparable with the results of another study by Book et al. in which DMARD treated patients were compared with the non-treated patients. In this study, BMD changes at both lumbar spine and femoral neck were not different with matched controls. In addition, the BMD values did not vary from baseline. 

Disease activity and disability were predictors of bone loss (15). In the present study, the changes in BMD Z-score at the femoral neck is consistent with the study of Kroot et al (16). In the latter study of over 8.9 years of treatment , the femoral neck BMD decreased by 0.28%±0.11 years but femoral neck BMD Z-score increased by 0.13±0.05 (16). The different levels of BMD changes between femoral neck and spine may be explained by the different pathogenetic mechanisms of bone loss between the femoral neck and lumbar spine regions. Inflammation is the most determinant of bone loss at the femoral neck (8) and so anti-rheumatic drugs and corticosteroids are expected to suppress femoral neck synovitis and exert beneficial effect in preserving further BMD. While the lumbar spine region is less affected by inflammatory process in RA, and therefore, no beneficial effect of anti-inflammatory treatment is expected to be gained. But in contrast, lumbar spine bone mass may be associated with decreased BMD due to exposure to high cumulative dose of corticosteroids. 

In another placebo-controlled study, treatment of RA for 3 years with DMARDS and prednisolone resulted in greater bone loss from lumbar spine compared with treatment without prednisolone, whereas, low dose MTX alone without prednisolone did not change femoral neck or lumbar spine BMD values (17). 

Several other factors of bone loss such as functional impairment, persistent of DA, aging and vitamin D deficiency may be considered as contributing factors of BMD reduction over the study period (18). Anti-inflammatory treatment decreases bone remodeling markers and so may be associated with lower bone resorption (6, 19). The findings of this study confirm the results of previous studies in regard to persistent bone loss during the treatment of RA with DMARDs (10, 15, 17, 20-24). In these studies, MTX had neither beneficial nor harmful effects (9, 21, 22). Reduction of BMD has been observed almost in all studies except in those studies where patients received DMARDs in combination with anti- antiresorptive treatment (20).

Our study has limitation in regard to small sample size, lack of control group and lack of data for other associated factors of BMD loss. The data presented here do not support any associated factors for bone loss in the study population. However, lower rate of bone loss at the femoral neck is in favor of inflammation as the main contributing factor of bone loss. However, disease duration and aging and inadequate attainment of peak bone mass in premenopausal stage, or physical disability should be also considered as a cause of reduced BMD during the treatment period of patients with RA (25). 

In conclusion, the findings of this study in agreement with earlier reports indicate that bone loss in RA is an ongoing phenomenon which continues despite anti-inflammatory treatment with DMARD. Preservation of bone mass in RA requires additional treatment program for osteoporosis.
